# Delineation of the population genetic structure of *Culicoides imicola* in East and South Africa

**DOI:** 10.1186/s13071-015-1277-4

**Published:** 2015-12-24

**Authors:** Maria G. Onyango, George N. Michuki, Moses Ogugo, Gert J. Venter, Miguel A. Miranda, Nohal Elissa, Appolinaire Djikeng, Steve Kemp, Peter J. Walker, Jean-Bernard Duchemin

**Affiliations:** CSIRO Health and Biosecurity, Australian Animal Health Laboratory, 5 Portalington Road, Geelong, VIC 3220 Australia; School of Medicine, Deakin University, 75 Pidgons Road, Waurn Ponds, VIC 3216 Australia; International Livestock Research Institute, P.O. Box 30709, 00100 Nairobi, Kenya; ARC-Onderstepoort Veterinary Institute, Private bag X5, Onderstepoort, 0110 South Africa; Laboratoria de Zoologia, University of the Balearic Islands, Palma de Mallorca, CP: 07122 Spain; Institut Pasteur of Madagascar, B.P. 1274 Ambatofotsikely, 101 Antananarivo, Republic of Madagascar; Biosciences eastern and central Africa – ILRI Hub (BecA-ILRI Hub), ILRI, PO Box 30709, 00100 Nairobi, Kenya

**Keywords:** *Culicoides imicola*, *Culicoides bolitinos*, Bluetongue virus, Mitochondrial DNA, Arboviruses, Population structure

## Abstract

**Background:**

*Culicoides imicola* Kieffer, 1913 is the main vector of bluetongue virus (BTV) and African horse sickness virus (AHSV) in Sub-Saharan Africa. Understanding the population genetic structure of this midge and the nature of barriers to gene flow will lead to a deeper understanding of bluetongue epidemiology and more effective vector control in this region.

**Methods:**

A panel of 12 DNA microsatellite markers isolated *de novo* and mitochondrial DNA were utilized in a study of *C. imicola* populations from Africa and an outlier population from the Balearic Islands. The DNA microsatellite markers and mitochondrial DNA were also used to examine a population of closely related *C. bolitinos* Meiswinkel midges.

**Results:**

The microsatellite data suggest gene flow between Kenya and south-west Indian Ocean Islands exist while a restricted gene flow between Kenya and South Africa *C. imicola* populations occurs. Genetic distance correlated with geographic distance by Mantel test. The mitochondrial DNA analysis results imply that the *C. imicola* populations from Kenya and south-west Indian Ocean Islands (Madagascar and Mauritius) shared haplotypes while *C. imicola* population from South Africa possessed private haplotypes and the highest nucleotide diversity among the African populations. The Bayesian skyline plot suggested a population growth.

**Conclusions:**

The gene flow demonstrated by this study indicates a potential risk of introduction of new BTV serotypes by wind-borne infected *Culicoides* into the Islands*.* Genetic similarity between Mauritius and South Africa may be due to translocation as a result of human-induced activities; this could impact negatively on the livestock industry. The microsatellite markers isolated in this study may be utilised to study *C. bolitinos*, an important vector of BTV and AHSV in Africa and identify sources of future incursions.

**Electronic supplementary material:**

The online version of this article (doi:10.1186/s13071-015-1277-4) contains supplementary material, which is available to authorized users.

## Background

Biting midges of the genus *Culicoides* (Diptera: Ceratopogonidae) are vectors of a number of arboviruses infecting livestock. Of the 75 arboviruses associated with *Culicoides*, 15 have been isolated from species belonging to *C. imicola* complex [1]. The *C. imicola* complex (*C. imicola*, *C. bolitinos*, *C. brevitarsis*, *C. nudipalpis, C. kwagga, C. Ioxodontis, C. miombo, C.pseudopallidipennis, C. tutti-frutti* and *C. asiatica*) [2, 3] contains the three most important known vectors of bluetongue virus: *C. imicola*, *C. bolitinos* and *C. brevitarsis.*

*Culicoides imicola* is widely distributed in Africa, the Mediterranean, India, Laos, Vietnam and southern China [1, 2, 4, 5]. It is capable of transmitting both bluetongue virus (BTV) and African horse sickness virus (AHSV) [5–7] and is considered to be the most important vector of these viruses in Africa [8]. Bluetongue disease was first described in 1903 in South Africa and was initially referred to as malarial catarrhal fever [9]. A total of 21 (numbered as 1–19, 22 and 24) serotypes have been identified in Africa [10]. South Africa currently has all 21 serotypes) [11]. Further north, in Kenya, 19 BTV serotypes have been isolated from sentinel cattle but clinical disease is not evident among the indigenous sheep [12]. A recent study in Madagascar revealed a very high prevalence of BTV serotype 2 in cattle and large distribution of the virus amongst domestic ruminants [13]. In 2003, the first outbreak of BTV (BTV-3) occurred in the neighbouring Island of La Reunion [14] and at least four serotypes (BTV-2, 17, 10 and 21) have been detected circulating in deer from Mauritius [15].

Presently, African horse sickness virus (AHSV) is endemic in tropical and sub-tropical areas of Africa south of the Sahara (East Africa, West Africa) extending as far south as the north of South Africa [16, 17]. Outside Africa, the disease is endemic in Yemen [17, 18]. However, the occurrence of AHS outbreaks in the Maghreb (western North Africa), then in Spain in 1965–66 and 1987–1990 indicates the epidemiological situation is fragile for non-endemic regions such as Europe or Madagascar and Mauritius [19]. In the absence of a wildlife reservoir, the trade-related movements of cattle and horse from one place to another is considered as the main driver of outbreak spread [20], but the hypothesis of *Culicoides* movement either passive or active cannot be ruled out. Because of their small size, *Culicoides* can be passively dispersed over long distance by prevailing winds [21]. Long distance wind dispersal of *Culicoide*s has been incriminated for the introduction of novel bluetongue virus serotypes and genotypes to new areas [22–24].

With such a rich diversity of bluetongue and AHSV serotypes circulating in the south and east of Africa, understanding the genetic structure of their vector *C. imicola* may give insights into the epidemiology of these diseases and further uncover possible transmission routes which could prevent future expansion into disease-free countries [25]. Given the geographical barriers (islands) within the south-west Indian Ocean region, we hypothesize a higher degree of restriction of gene flow between the islands and the continental mass than within the continent.

Despite its importance as a vector, there is scarce information about the population genetic structure of *C. imicola* in Africa. Sebastiani et al. [26] demonstrated molecular differentiation of the old world *C. imicola* species complex from southern Africa, Madagascar and the Ivory Coast. Using random amplified DNA (RAPD) markers, polymorphic bands that resulted in species-specific RAPD profiles were used to carry out molecular analysis of variance (AMOVA) test in order to assess the allelic variation between and within the species populations. A high level of intra-population genetic differentiation and a relatively lower level of inter-population genetic differentiation were observed. Madagascar populations of *C. imicola s.s.* revealed isolation by distance pattern. Mardulyn et al. [27] study used microsatellite markers to study genetic variation among *C. imicola* populations from southern Europe and North Africa and performed approximate Bayesian computation framework analysis on the allelic variation obtained by microsatellite genotyping. The findings supported a weak population structure between northern Africa *C. imicola* populations and southern Europe populations of *C. imicola* that led to the conclusion of a possible ancient existence of *C. imicola* in Europe.

Utilizing the same mitochondrial and microsatellite markers developed by Mardulyn et al. [27], Jacquet et al. [28] have also demonstrated the ancient existence of *C. imicola* in Europe and the possible routes of incursions of *C. imicola* into Europe from Africa. Using the mitochondrial gene marker *cytochrome oxidase I* (*COI*) Desvars et al. [29] found a lack of genetic connectivity between *C. imicola* populations from Reunion Island, Spain and South Africa.

This study was designed to provide quantitative data about genetic differentiation of the *C. imicola* populations within the East and South regions of Africa. It has considered the potential geographical and physical factors, acting as barriers or motors to gene flow and dispersal. Technically, the first aim of this study was to develop de novo DNA microsatellite markers for *C. imicola* using a recently described experimental workflow [30]. The second aim of the study was to employ both the developed microsatellite markers and DNA sequences of the *COI* gene to test genetic diversity amongst geographically different populations from Balearic Islands, Kenya, South Africa, Madagascar and Mauritius. The third aim was to utilise the novel microsatellite markers designed for *C. imicola* to amplify microsatellite repeats of the closely related species *C. bolitinos*, an important vector of BTV and AHSV in sub-Sahara Africa.

## Methods

### Collection of midges

**Fig. 1:**
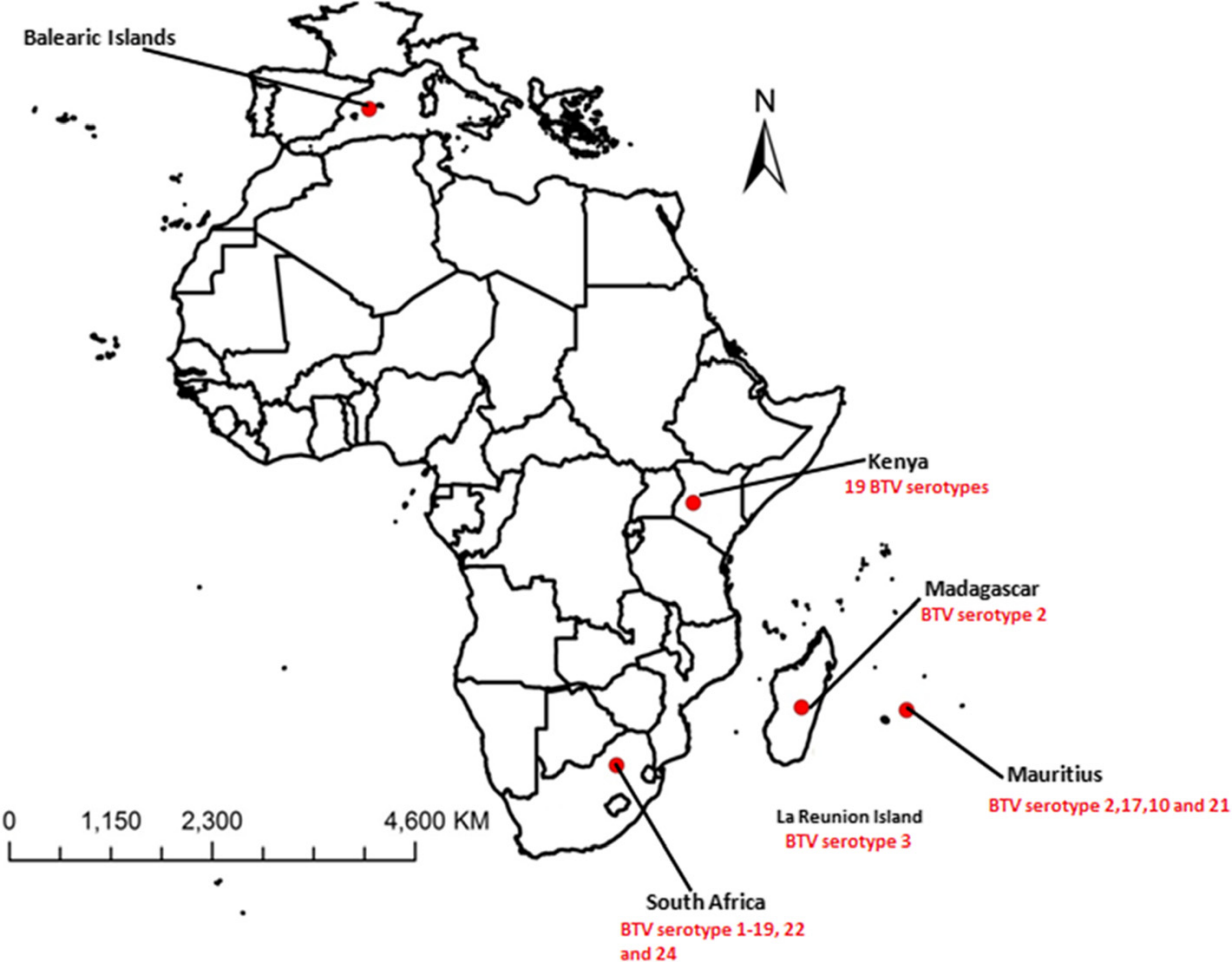
Map showing *Culicoides imicola* collection sites and serotype distribution in Africa . Map source: Arc Map

The collection sites for midges used in this study are shown in Fig. 1 and Table 1. A total of 138 *C. imicola* midges were collected from the Balearic Islands, Kenya, South Africa, Madagascar and Mauritius and 5 samples of *C. bolitinos* midges were collected from Madagascar. Samples of both *C. imicola* and *C. bolitinos* were collected using traps set at night 1 h before sunset to around 0800 h at dawn. The samples were transported to the laboratory in 70 % ethanol. Morphological identification was based on the wing patterns [31] observed using a binocular microscope. Species identification was confirmed by amplification of the *mt*DNA *COI* gene [2] to ensure exclusion of morphologically similar species.

**Table 1:** A table of the sites of midges collection with the locations and global positioning system coordinates of *Culicoides imicola* samples from Spain and Africa. The collection method used was the CDC light trap

Species	Region	Site	Latitude	Longitude	n (Microsatellite)	n (Mitochondrial DNA)
*C. imicola*	Kenya	Marigat	0.47	35.99	26	21
*C. imicola*	Balearic Islands	Mallorca	39.61	2.95	24	14
*C. imicola*	Madagascar	Ambalavao	−21.83	46.93	25	17
*C. imicola*	Mauritius	Poste Lafayette	−20.14	57.75	35	13
*C. imicola*	South Africa	Onderstepoort	−25.65	28.18	28	13
*C. bolitinos*	Madagascar	Ambalavao	−21.83	46.93	5	5

### *Cytochrome oxidase I* (*COI*) gene amplification and sequencing

The *mtDNA COI* gene was amplified from 83 individuals (Kenya, South Africa, Madagascar, Mauritius and Balearic Islands) [GenBank numbers: KT339684- KT339742; KT945260-KT945271; KT968152- KT968163] using primer pair *Bc1 Culic Fm* (GTA AAA CGA CGG CCA GTT CWA CWA AYC AYA AAR WTA) and *JerR2m *(CAG GAA ACA GCT ATG ACC CAA ARA ATC ARA AYA RRT GTT G) [32]. The amplification was carried out under the following conditions: Initial denaturation of 94 °C for 2 min, 40 cycles of 94 °C for 30 s, 50 °C for 30 s, 72 °C for 1 min and a final elongation step of 72 °C for 5 min. Each 25 μl PCR reaction included 1.0 μl template DNA (20 ng), 0.2 μmol each of forward and reverse primer, 18 μl Platinum PCR supermix high fidelity (Life Technologies) and 2.75 μl de-ionized water. The PCR amplicons were purified using QIAquick PCR purification kit (Qiagen) and 20 μl was sequenced using the Sanger sequencing method (Macrogen, Geumchun-gu, Seoul).

### Phylogenetic and demography history analysis of mitochondrial DNA *(mtDNA)*

The sequences were aligned using MUSCLE [33] and then trimmed to a uniform length. A total of 11 previously identified and published haplotypes (Greece and Israel) were added with the following GenBank accession numbers: [AF078098 - 100, AF080531-2, AF080534, AJ549388-92] [6, 34]. To examine phylogenetic relationships, haplotype networks were constructed in PopART [35] using TCS network (95 % connection limit). The historical demographical processes were inferred using several methods. Haplotype diversity (h), nucleotide diversity (π) and neutrality tests (Fu’s F’s and Tajima’s D) were computed in Dna Sp v.5 [36] and Arlequin v 3.11 [37].

A coalescent-based Bayesian skyline plot was utilised [38] to estimate the effective population size. This method uses the Markov chain Monte Carlo (MCMC) procedure to estimate the posterior distribution of effective population size. The analysis was conducted using the HKY substitution model with empirical base frequencies, with each codon having its own rate of evolution and a strict clock model enforced. A total of 10 000 000 iterations were run, a burn-in of 1 000 000, while the parameters of the model were stored every 1 000 iterations. The Bayesian phylogenetic analysis were carried out using BEAST v1.8.2 [38]. The posterior distributions and the graph were plotted using TRACER v1.6 software.

### Isolation and validation of microsatellites markers

*De novo* isolation of microsatellite DNA was carried out using a recently-developed workflow [30]. In brief, genomic DNA was obtained from four pools of midges (each pool consisting of 15 samples) from Kenya, Madagascar, Balearic Islands and South Africa using the DNeasy blood and tissue kit (QIAgen) according to the manufacturer’s protocol and was quantified using a Qubit fluorometer (Life Technologies, Invitrogen).

The pools of genomic DNA were whole genome amplified (WGA) using the REPLI-g midi kit (QIAgen) according to the manufacturer’s protocol. Each pool of WGA DNA was subjected to whole genome sequencing on a Roche 454 Genome Sequencer FLX. The raw reads of the WGA DNA sequence were screened directly for microsatellite repeats using MSATCOMMANDER v0.8.2 [39]. Primers flanking the microsatellite repeats were designed using the “Design Primers” option. The sequences of the designed primers were aligned to test for possible identical PCR primer annealing sites.

Initially, a total of 20 primer pairs flanking dinucleotide repeats were selected for validation. These primers were used to amplify 12 individual midge samples (consisting of three individuals from each country) from Kenya, Balearic Islands, Madagascar and South Africa. Samples of *C. bolitinos* midges were also tested for cross-species amplification by these markers. The primers that amplified 100 % of the subpopulation were tagged at the 5’ end with one of the fluorescent dyes FAM, HEX or NED (Table 2). Loci with sufficiently different size ranges were labeled with same dye while those with similar size ranges were labeled with a different dye allowing co-loading of the samples.

**Table 2:** *Culicoides imicola* microsatellite primer sequences developed and assessed in this study

Locus name	Left primer	Right primer	NA	Size range	Motif type	Probe DB
F1L88	GTCGGTTGGTGTGTGTCATC	ACGACGACATTATTGACAGCAG	16	180–286	(GT)^10	Pr032368558
F2629	ACTCTCAAGGTTTCCGCTG	GACGGCAAACAAAACATGCC	20	130–242	(GT)^9	Pr032368559
F2KK2	ACGTGGTACTCAAAGGCAG	CCATGTGATACAGCTTGCGG	21	166–228	(GT)^9	Pr032368560
F3JMT	CCGATAGTTGTTGTCGTTCATTC	GTATGAGACTCGGTTTGCAC	10	212–274	(GT)^8	Pr032368561
F7ENT	CTGCCTTTTCCACCTCCAC	ATGCCAGAGTGAAAGCGAC	12	94–198	(AC)^9	Pr032368562
F9RDN^b^	AACAAAACACAGCCGCGAG	ATCAGCCAGTCCGCATAAG	16	134–246	(AC)^9	Pr032368563
FHNDU	ACGGGTCCGTGTATTTGTTG	GACGAGATACGGGCGAGAG	14	172–198	(AC)^8	Pr032368564
FIGO0^a^	CAGCAATAAATTGTGTGTCATAACC	GCTTCTCACTCTCCAAACATCTG	15	184–226	(GT)^8	Pr032368565
FJEAX	TCACGCCTGAACATGGGAG	AACAACAACAGAGGCAGGC	11	185–210	(GT)^8	Pr032368566
FRB3B	TCCAGCCATCGTCTTTCAG	GGGTGTGTGTAAACTCTATTGTAGC	15	198–234	(AG)^8	Pr032368567
FYCEH	CGCCACGCCATTTATCGTC	ACTGACAGCTTCCTCTCGC	26	108–248	(AC)^12	Pr032368568
G1OH4	TCTCCCAGAAGCGTTTTGC	GTCGTCGTTCTGCCTTGC	11	98–268	(AC)^8	Pr032368569

^a^ discarded as it was in linkage disequilibrium with loci FHNDU (P < 0.05). ^b^excluded from downstream analysis because of too much missing data

### Microsatellite analysis

A total of 143 individuals (Table 1) were genotyped at 12 microsatellite DNA loci (Table 2). Each 25 μl PCR reaction included 1.0 μl template DNA (20 ng), 0.2 μmol (each) forward and reverse fluorescent labelled primer (Applied Biosystems, USA), 18 μl Platinum PCR supermix high fidelity (Life Technologies) and 2.75 μl de-ionized water (Life Technologies). The amplification was carried out under the following conditions: initial denaturation of 94 °C for 3 min, then 14 cycles of 94 °C for 30 s, 59 °C for 30 s with a gradient decrease of 1 °C/cycle, 72 °C for 30 s followed by 30 cycles of 94 °C for 30 s, 46 °C for 30 s, 72 °C for 30 s and a final elongation step of 72 °C for 7 min. Amplified PCR products were fragment-sized by an external contractor (Macrogen, Geumchun-gu, Seoul). The fragment lengths were analysed and corrected manually using Peak Scanner v2.0 (Applied Biosystems). A fraction (5 %) of the original samples was re-run as a validation of initial allelic scores.

### Data analysis

Genepop [40] was used to test for linkage disequilibrium between each pair of loci and across regions (Fischer’s method) and to estimate allelic diversity and the coefficient of inbreeding (F_IS_) at individual loci within populations. Microchecker [41] was used to check for putative null alleles, large allele dropout or stutter peaks.

Departure from the Hardy-Weinberg equilibrium for each locus within each population and a global test across all loci were carried out using Arlequin v3.1 [37]. The observed numbers of heterozygotes and homozygotes at loci in each population were tested against the expected numbers using a chi-square test.

Multilocus genetic distance [42] and fixation index (F_IS_) [37, 43] estimates were calculated between population pairs using Genepop and Arlequin [37, 40] in order to describe the genetic structure of the populations from the microsatellite data. Permutation tests (100 replications) were used to determine the significance of the population structure estimates.

A model-based clustering method [44] was used to infer population structure probabilistically and assign individuals to populations using the microsatellite data, as implemented in the Bayesian program STRUCTURE v2.3.4 [44]. A total of 22 independent runs and a K value range from 1–10 of the total data sets were carried out. A burn-in period of 100,000, Markov chain Monte Carlo (MCMC) repeats of 100,000, ancestry model of admixture and LOCPRIOR model that used sampling location as prior information were applied to assist clustering. Because replicating STRUCTURE runs creates stochastic effects, resulting in different outcomes, simplifying the assessment of replicate data by calculating medians is important. CLUMPAK [45] was used to collate all the data into a matrix (the Q matrix) of individual membership co-efficient and population ancestry components. CLUMPAK utilizes the LargeKGreedy algorithm of CLUMMP. A total of 2000 repeats were used in this study. STRUCTURE HARVESTER v0.6.94 [46] was used to infer the most likely number of genetic clusters (K) present using both the Evanno and Delta K methods.

The IBD program [47] was used to test for correlation between the pairwise genetic distance (F_ST_) and the geographic distance matrix with a Mantel test. The statistical significance of this correlation was determined using reduced major axis (RMA) regression, a method specifically formulated to handle errors in the x and y variables to reduce bias in estimates of slopes. Error estimates were calculated using three methods: standard linear regression approximations, jackknifing over cases and bootstrapping over cases. Genetic distance was once more regressed against geographic distance omitting the Balearic Islands population that served as an outgroup.

## Results

### *Cytochrome oxidase I* (*COI*) gene analysis

**Fig. 2:**
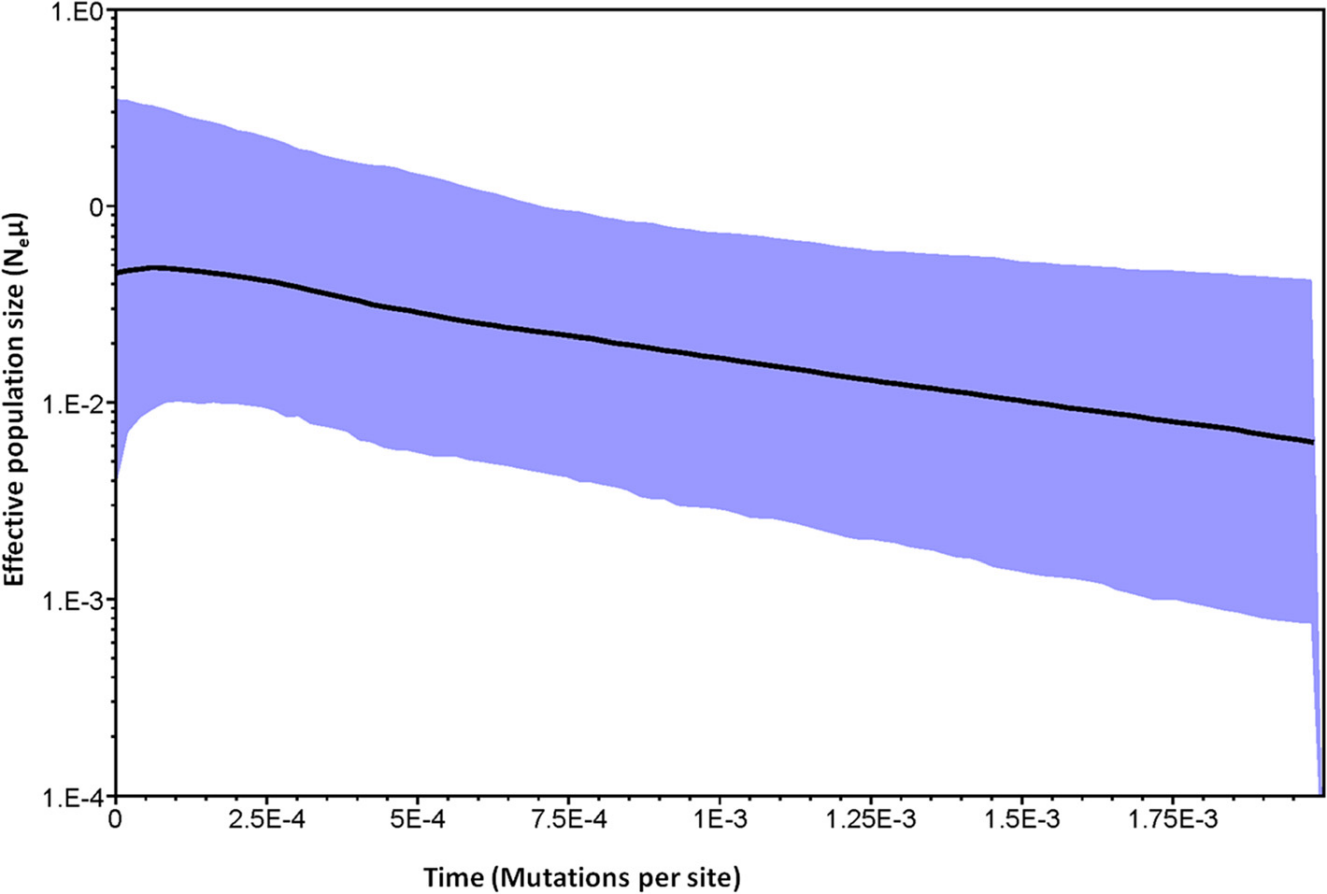
Bayesian skyline plot based on partial sequences of the mitochondrial region. X axis represents time measured in mutation units per nucleotide position. The Y axis represents a correlate of population size Neμ). Black lines illustrate median Ne estimate, and blue area show the 95 % confidence interval

A total of 27 *mt*DNA *COI* haplotypes were identified in the 94 sequences from both *C. imicola* and *C. bolitinos* (Hd = 0.8490) (Table 3). Neutrality tests yielded negative values Fu and Li’s F statistic = −2.18511 and Tajima’s D = −1.52771 but were statistically insignificant (P > 0.05). South Africa, Balearic Islands and Kenya had statistically significant (*P* < 0.05) negative FS value while Mauritius and Kenya had statistically significant (P < 0.05) negative Tajima’s D value (Table 4). The Bayesian skyline plot suggested population growth (Fig. 2).

**Table 3:** A summary of *mt*DNA COI study collection sites and haplotype distribution

Region	Haplotype																											
	H1	H2	H3	H4	H5	H6	H7	H8	H9	H10	H11	H12	H13	H14	H15	H16	H17	H18	H19	H20	H21	H22	H23	H24	H25	H26	H27	Totals
South Africa	1																3	1	3	1	1	1	1	1				13
Madagascar			10	4		1																						15
Kenya			12	2	2	1			1	2	1	1			1													23
Israel					5																				1			6
Balearic Islands							13	1																				14
Greece																										2	3	5
Mauritius		1	11													1												13
*C. bolitinos*													1	4														5
Total	1	1	33	6	7	2	13	1	1	2	1	1	1	4	1	1	3	1	3	1	1	1	1	1	1	2	3	94

**Table 4:** A table of estimates of Tajima’s D neutrality test of COI haplotypes *C. bolitinos* and *C. imicola*

	South Africa	Mauritius	Madagascar	Balearic Islands	Kenya	Israel	Greece	Mean	S.D
Sample size	13	13	17	14	21	6	5	11.75	5.9
S	10	3	3	1	8	2	3	8.6	12.7
π	2.5	0.5	0.72	0.14	1.2	0.7	1.8	2.9	5.2
Tajima’s D	−0.91	−1.7	−0.53	−1.2	−1.7	−1.13	1.57	−0.78	1.11
P(Tajima’s D)	0.19	**0.02**	0.32	0.16	**0.02**	0.14	0.97	0.26	0.33
FS	−4.3	−0.69	−0.92	−0.59	−4.06	0.95	2.43	−1.03	2.45
P(FS)	**0**	0.08	0.15	**0.02**	**0**	0.61	0.85	0.24	0.34

Bold indicate the significant values

**Fig. 3:**
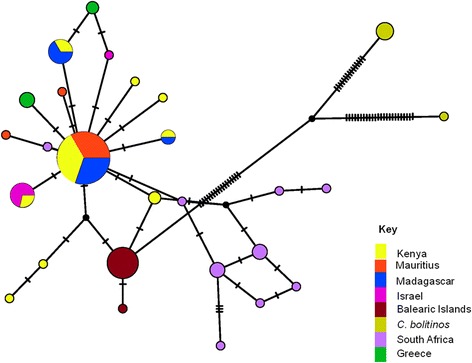
Haplotype networks of *C. imicola* COI sequences from the Mediterranean, Africa and *C. bolitinos* from Madagascar. Each circle represents a sequence; the size of the circle is proportional to number of individuals in possession of the particular haplotype sequence. The connections are mutational steps between individuals

The haplotype network showed a complex and moderately diversified topology. The network consisted of one main star-like arrangement with satellites and a more complex adjacent network (Fig. 3). The main core group consisted of the East African and south-western Indian Ocean Islands populations associated with eastern Mediterranean *C. imicola* haplotypes (Greece and Israel). Except for a single haplotype directly linked to the main star-like haplotype, the South Africa population consisted mainly of private haplotypes differing by a few mutations and is organised in an adjacent network. At the intermediate position, the eastern Mediterranean population (Balearic Islands) possessed private haplotypes and so did the *C. bolitinos* population from Madagascar that served as an out-group (Table 3).

### Isolation of microsatellite repeats

A total of 1450 putative microsatellite repeats were isolated from 367210 reads sequenced. Of these putative microsatellites, 1189 were dinucleotide, 181 were trinucleotide and 80 were tetranucleotide repeats. A total of 465 primers were designed on the flanking regions of the microsatellite repeats. Of these, 249 were found to be similar to each other or blasted to an existing organism in the database and so were considered to be products of contamination. The remaining 216 unique primer pairs were subsequently blasted against the sequenced raw reads. Of these, 169 primer pairs matched a single read (Additional file 1) and 34 primer pairs matched either more than one read or more than one site on a single read. After reverse-complementing the right primer, the primer pairs that were found to match more than one read were aligned to the matching reads. Upon checking the primer point of annealing on individual reads, it was evident that primers matching more than one read were either not properly positioned on the microsatellite repeat flanking regions or matched sequences outside of the repeat regions. These primer pairs were excluded from the study.

### Intra- and inter-population diversity, Hardy-Weinberg equilibrium and linkage disequilibrium

Substantial variation was observed among the 12 microsatellite loci used to genotype the individual *C. imicola* midges (Table 2 and Additional file 2). Exact tests for linkage disequilibrium showed a significant association between the loci FIGOO and FHNDU. The locus FIGOO was discarded, as it did not result in further linkage disequilibrium. Locus F9RDN was also discarded because it resulted in high null amplification (19 %). A total of 187 alleles were scored ranging from 11–26 alleles/locus (Table 2). Initial tests conducted for each locus in all populations indicated statistical significance of departure from Hardy-Weinberg equilibrium (P < 0.05) in 71 % of the instances. The expected genetic diversity (H_e_) varied from 0.2 to 0.94 while the observed heterozygosity (H_o_) ranged from 0.125 to 1.0. Putative null alleles were identified at some loci in some populations using Microchecker. The null alleles were not locus-specific (Additional file 2). To check for congruence in the results, a total of 77 DNA samples that were initially identified either as homozygotes at one or more of the 12 loci or had failed to amplify were re-amplified using a touchdown PCR program. Two samples (3 %) failed to successfully re-amplify, ten samples (13 %) initially scored as homozygous were scored as heterozygous, six samples (8 %) initially scored as heterozygotes were scored as homozygotes while 17 samples (22 %) scored as null were successfully re-amplified and 42 samples (56 %) were un-altered. A third run was carried out to amplify all the individuals that failed at the two previous attempts.

### Population differentiation and isolation by distance (IBD)

**Fig. 4:**
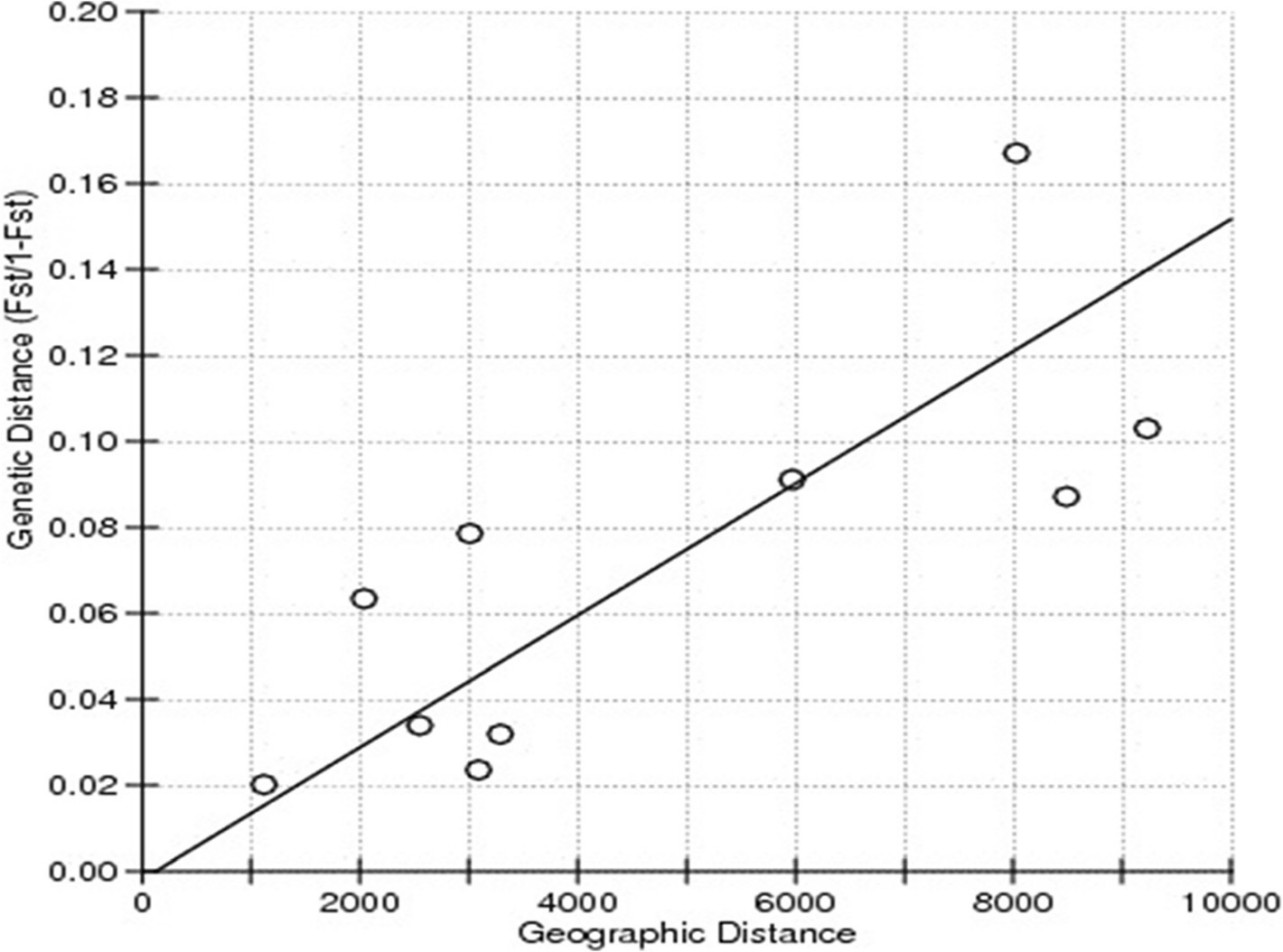
Mantel test for correlation between the pairwise genetic distance and the geographic distance matrix. Regression analysis of pairwise genetic distance (F_ST_/ 1- F_ST_) regressed on pairwise geographic distance (Km) between collection sites revealed a significant positive correlation (r^2^ = 0.607, mantel probability = 0.0079 (*P* < 0.05)

Statistically significant genetic differentiation (*P* < 0.05) was demonstrated between the Balearic Islands population and all the other populations [Balearic Islands vs. Madagascar (F_ST_ = 0.08), Balearic Islands vs. Mauritius (F_ST_ = 0.09), Balearic Islands vs. South Africa (F_ST_ = 0.14), Balearic Islands vs. Kenya (F_ST_ = 0.08)]. The rest of the African populations seemed to have no genetic differentiation except between the Kenyan population and South African (F_ST_ = 0.07) and South Africa and Madagascar populations (F_ST_ = 0.06) in the tests employed (Table 5). F_ST_ estimates varied from 0.02 to 0.14. In spite of being geographically closer (approximately 2040 km) the F_ST_ value of the South Africa and Madagascar populations was higher (F_ST_ = 0.06) than the F_ST_ value between the South Africa and Mauritius populations (F_ST_ = 0.02) (approximately 3090 km) (Table 5). The results of the Mantel test for correlation between genetic distance and geographic distance matrix supported a significant positive correlation (r^2^ = 0.607, Mantel probability *p* = 0.0079) (Fig. 4). The null hypothesis of no correlation between geographical and genetic distance was therefore rejected. When the correlation test was re-run after omitting the Balearic Islands population, a non-significant positive correlation did not support IBD effect.

**Table 5:** A matrix of pairwise estimates of genetic distance (F_ST_) (below diagonal) and geographic distances (Km) (above diagonal) of microsatellite DNA of *C. imicola* populations from Balearic Islands and Africa

	Kenya	Balearic Islands	Madagascar	Mauritius	South Africa
Kenya		5970	2550	3290	3010
Balearic Islands	**0.08(0.00 + −0.00)**		8490	9230	8030
Madagascar	0.03(0.00 + −0.00)	**0.08(0.00 + −0.00)**		1120	2040
Mauritius	0.03(0.00 + −0.00)	**0.09(0.00 + −0.00)**	0.02(0.00 + −0.00)		3090
South Africa	**0.07(0.00 + −0.00)**	**0.14(0.00 + −0.00)**	**0.06(0.00 + −0.00)**	0.02 (0.00 + −0.00)	

Bold values are statistically significant (*p*<0.05)

### Bayesian analysis of population differentiation

**Fig. 5:**
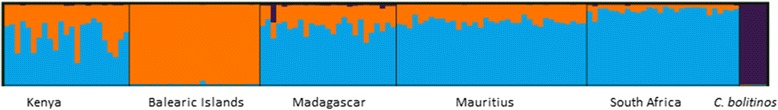
Plots of cluster assignment of individuals into three founder populations. Each individual is represented by a single vertical line, partitioned into K-colored segments that represent the individual’s estimated membership fraction in each of the inferred cluster

Evanno and Delta methods identified the most probable clustering values of K = 3 (Fig. 5). The coloured columns in the matrix show the inferred ancestry membership proportional probabilities of each individual. The posterior probabilities suggested that *C. bolitinos* individuals were not admixed. As a whole, *C. imicola* populations demonstrated admixture. However, the Balearic Islands and *C. bolitinos* population showed a very distinct pattern.

## Discussion

Long distance wind dispersal of *Culicoide*s has been incriminated for the introduction of novel bluetongue virus serotypes and genotypes to new areas [22–24]. Because of their small size, *Culicoides* can be passively dispersed over long distance by prevailing winds [21]. The microsatellite results of this study revealed gene flow patterns between Kenya and south-west Indian Ocean Islands (Madagascar and Mauritius) (F_ST_ = 0.03) (Table 5) which could be due to passive wind-blown *Culicoides* by the south-west monsoon winds travelling northwards from the South-west Indian Ocean Island to the Kenyan Coast from April/May to October [48] while the northeast monsoon winds blowing south from the Kenyan coast from December to mid-March could blow the midges to the Islands. Despite a shorter distance between Madagascar and South Africa, the populations show a lower degree of gene flow probably due to the orientation of monsoon winds flow being away from South Africa [48]. However, the Mauritius and South Africa *C. imicola* populations seemed genetically similar (F_ST_ = 0.02) (Table 5). This could underpin alternative ways of dispersal of the vector. Movement due to human activities, especially international animal trade, cannot be ruled out. At a different scale (continental) and using a different set of microsatellite markers [27], Jacquet et al. [28] found F_ST_ values similar to our study and highlighted a strong differentiation between and within the Meditteranean and African populations of *C. imicola*.

The lack of genetic homogeneity between the Kenyan and South African populations of *C. imicola* as shown by microsatellite results (F_ST_ = 0.07) (Table 5) and the shape of the haplotype network of COI sequences, suggest limits to gene flow. Physical and/or anthropogenic barriers could occur. The spatial distribution of *C. imicola* is highly dependent on soil type and soil moisture for breeding sites, in habitats open to sunlight [5, 49]. A similar pattern was observed for *Anopheles gambiae* Giles and *Anopheles funestus* Giles in Africa in which population structures resulting in split between lineages are thought to have arisen due to extreme past droughts in East Africa [50, 51].

A phylogenetic study of *C. brevitarsis,* another member of the Imicola Complex, in the Australasian region showed no evidence of genetic separation or structure between populations sampled from northern and eastern Australia, approximately 3000 km apart, suggesting a panmictic population in the continent [30]. The breeding habitat of *C. brevitarsis* is restricted to fresh dung of wild and domesticated bovids [52]. Its distribution in Australia, within its climatic niche, coincides with the distribution of cattle [53] suggesting that this species of biting midge could not have existed in Australia before the introduction of cattle to the continent in the late 19th Century [54]. Therefore, the genetic connectivity that is evident among the populations of *C. brevitarsis* within Australia could be an indication of a recent colonization and expansion into this region [30]; this is in contrast to probably longer past history of *C. imicola* in South Africa and Kenya.

Model-based clustering method used to infer structure in this study identified that three-cluster was most probable. The K = 3 model scenario suggests a single homogenous population of the African *C. imicola* populations sampled and genetically distinct Balearic Islands and *C. bolitinos* populations.

This study has revealed a significant positive correlation between genetic distance and geographic distance for *C. imicola*, indicative of IBD pattern. Isolation by distance, initially described by Wright [55], describes an accumulation of local genetic differences under geographically restricted dispersal [56]. Thus, neighboring populations exchange more migrants than the distant ones, resulting in a significant decline in gene flow with distance. Previous population genetic studies have reported a fine scale IBD pattern among damselflies brought about by their sedentary nature which leads to restricted movements and localized breeding [57], while Lehmann [50] found that distance contributed minimally to the IBD pattern in the African populations of the malaria vector, *Anopheles gambiae*. In the present study, the IBD pattern disappears when the geographically outlying population of Balearic Islands is excluded, suggesting that the pattern may be due to vicariance resulting from reduced gene flow between the geographically outlying Balearic Islands population and the southern and eastern African populations of *C. imicola* that we have sampled [58].

The microsatellite markers isolated in this study were variable. The departure from Hardy-Weinberg equilibrium observed in this study could be as a result of presence of null alleles caused by mutations at locus-specific primer annealing sites, resulting in an excess in homozygosity [59].

The effect of null allele on genetic test of population structure has been shown to result in small upward biases to F_ST_ (0.003–0.004) and slight reduction in the power of STRUCTURE to correctly assign individuals to populations (0.2 and 0.1 % units) [60]. With these caveats, we argue that the likely slight upward biases in population genetic structure parameters and specimen cluster assignments using STRUCTURE will not drastically alter the results of our study.

Twelve microsatellite markers isolated in this study from the *C. imicola* genome were also detected in our few *C. bolitinos* individuals. All the loci were amplified and the alleles were quite varied. This species is more closely related phylogenetically to *C. brevitarsis* and could be a better vector of BTV than *C. imicola* [61]. The development of genetic markers that are capable of deciphering the genetic structure of this species will be very useful in studying their populations in the African region.

The mitochondrial *COI* gene haplotype network corroborated to a great extent the microsatellite results. Kenya and south-west Indian Ocean Islands (Mauritius and Madagascar) shared haplotypes. Except for a single haplotype which was directly linked to the main haplotype, South Africa population mainly possessed private haplotypes. This could be indicative of a contemporary only modest matrilineal gene flow between east and south of Africa. The Kenyan and south-west Indian Ocean populations shared haplotypes with the eastern Mediterranean populations of *C. imicola* (Israel and Greece). The western Mediterranean population (Balearic Islands) had private haplotypes. This could be indicative of different routes of *C. imicola* dispersal in the Mediterranean region [62] as proposed by Jacquet et al. [28] . However, a more detailed and extensive sampling and multiple loci population genetic study would be required to substantiate this claim.

The Bayesian skyline plot suggested a population growth, compatible with the star-like pattern of the Kenya/south-west Indian Ocean Islands mitochondrial marker haplotype network and the Tajima’s D and Fu’s F test values for Kenya, South Africa and Mauritius. This could be indicative of a recent increase in favourable conditions that increased suitable habitats but also expansions of its distributions in territories where bovids and equids, domesticated or wild were absent for a long time or changing farming practices cannot be ruled out. However the Bayesian skyline plot ought to be interpreted with caution as Bayesian skyline plot appears to slightly overestimate the most recent population [38]. More extensive analytical procedures need to be employed in order to prove this claim.

This study showed a higher frequency of private haplotypes among the South African *C. imicola* population as well as the highest nucleotide diversity. This may suggest an ancestral history, reinforced by the closely related species described from the region [63–65]

## Conclusions

The findings of this study demonstrate notable risk of movement of *Culicoides*- borne virus within the East and South regions of Africa, especially between Kenya and neighboring islands (Mauritius, Madagascar), demonstrated by two types of genetic markers and analysis. This risk is probably due to passive wind-borne infected *Culicoides*, implicating the monsoon winds in the Indian Ocean. Such vector midge movement could impact negatively on local livestock production and equids industry. On the other hand, we found a reduction in *Culicoides* genetic flow within the continental mainland, between South Africa and Kenya. The microsatellite markers isolated in this study could be applied in genotyping *C. bolitinos*, another important vector of BTV and AHSV in the region.

## Additional files

Additional file 1:
**A summary of the** ***C. imicola*** **markers designed to the flanking regions of the microsatellite repeats with the type of repeats.** The table provides a list of a total of 169 primer pairs predicted to anneal to a unique single pair read. The motif type that the primer represents is also indicated (XLS 41 kb) Additional file 2:
**A table of estimates of genetic diversity of the microsatellite markers per population.** The table describes the genetic diversity of the markers. Significant Hardy-Weinberg equilibrium (*P* < 0.05) tests are in BOLD. The expected gene diversity (H_e_) varied from (0.2–0.94) while the observed heterozygosity (H_o_) ranged from (0.125–1.0). Putative null alleles were identified at some loci in some populations using Microchecker. The null alleles were not locus specific. H_o_- Observed heterozygosity; H_e_- Expected heterozygosity; P_HWE_- Probability of Hardy-Weinberg equilibrium; F_IS_- Inbreeding coefficient; N_allele_- Null allele; n- number of Samples (XLS 29 kb)
